# Transcutaneous Administration of Dengue Vaccines

**DOI:** 10.3390/v12050514

**Published:** 2020-05-06

**Authors:** Robert Andreata-Santos, Rúbens Prince dos Santos Alves, Sara Araujo Pereira, Lennon Ramos Pereira, Carla Longo de Freitas, Samuel Santos Pereira, Alexia Adrianne Venceslau-Carvalho, Maria Fernanda Castro-Amarante, Marianna Teixeira Pinho Favaro, Camila Mathias-Santos, Jaime Henrique Amorim, Luís Carlos de Souza Ferreira

**Affiliations:** 1 Vaccine Development Laboratory, Microbiology Department, Institute of Biomedical Sciences, University of São Paulo, São Paulo 05508-000, Brazil; robert_andreata@hotmail.com (R.A.-S.); rubens.bmc@gmail.com (R.P.d.S.A.); araujopereirasara@gmail.com (S.A.P.); lennon_rp@hotmail.com (L.R.P.); carla.longofreitas@gmail.com (C.L.d.F.); samuelbiomedicina@usp.br (S.S.P.); alexia_myt@hotmail.com (A.A.V.-C.); mfamarante@gmail.com (M.F.C.-A.); favaro.mtp@gmail.com (M.T.P.F.); camilamathias@gmail.com (C.M.-S.); 2 Center for Biological and Health Sciences, Federal University of Western Bahia, Bahia 47810-047, Brazil; jaime.henrique.amorim@gmail.com

**Keywords:** transcutaneous immunization, dengue vaccines, heat-labile toxin, adjuvant, intradermic immunization

## Abstract

In the present study, we evaluated the immunological responses induced by dengue vaccines under experimental conditions after delivery via a transcutaneous (TC) route. Vaccines against type 2 Dengue virus particles (DENV2 New Guinea C (NGC) strain) combined with enterotoxigenic *Escherichia coli* (ETEC) heat-labile toxin (LT) were administered to BALB/c mice in a three-dose immunization regimen via the TC route. As a control for the parenteral administration route, other mouse groups were immunized with the same vaccine formulation via the intradermic (ID) route. Our results showed that mice vaccinated either via the TC or ID routes developed similar protective immunity, as measured after lethal challenges with the DENV2 NGC strain. Notably, the vaccine delivered through the TC route induced lower serum antibody (IgG) responses with regard to ID-immunized mice, particularly after the third dose. The protective immunity elicited in TC-immunized mice was attributed to different antigen-specific antibody properties, such as epitope specificity and IgG subclass responses, and cellular immune responses, as determined by cytokine secretion profiles. Altogether, the results of the present study demonstrate the immunogenicity and protective properties of a dengue vaccine delivered through the TC route and offer perspectives for future clinical applications.

## 1. Introduction

Infection with one of the four Dengue virus serotypes (DENV1-4) may cause a spectrum of diseases ranging from an acute, self-limiting febrile illness (DF) characterized mainly by fever, retro-orbital headache, rash, arthralgia, and to more severe, life-threatening, conditions that may include hemorrhagic manifestations, increased vascular permeability, thrombocytopenia, and shock [1,2,3]. In fact, it is estimated that 3.9 billion people in 128 countries are at risk of infection [2,4]. DENV causes approximately 390 million infections, of which 500,000 cases develop into severe forms, making DENV infection one of the most economically and epidemiologically relevant arthropod-borne diseases transmitted to humans [2,4]. At present, there is no antiviral therapy or vaccine that is safe, effective and widely available.

A long-term solution against the global DENV challenge is the development of a safe, cost-effective, and efficient vaccine. In this sense, the administration route may have relevant impacts on the induced immune responses by a given vaccine formulation. The transcutaneous (TC) immunization route enables the delivery of soluble or particulate antigens on scarified naked skin with the aid of adhesive patches, thus avoiding the use of needles and syringes [5,6,7,8]. The epidermis is an anatomic site with a high concentration of specialized antigen-presenting cells (Langerhans cells) that can efficiently process antigens at draining lymph nodes and present epitopes to effector cells, such as B and T cells, which subsequently trigger adaptive long-lived antiviral immune responses [9,10,11,12,13]. In fact, viral vaccines containing antigens of hepatitis B virus, human immunodeficiency virus (HIV), Japanese encephalitis virus and herpes simplex virus successfully induced effective antigen-specific antibody responses after TC immunization in mice [14,15,16,17,18,19]. However, alternative immunization sites, such as those based on mucosal or the TC routes, have not been the particular focus for testing anti-DENV vaccines. Moreover, limited information is available concerning the induction of protective immunity against DENV infection after immunization via alternative administration routes.

In the present study, we tested the administration of an anti-DENV vaccine formulation delivered via the TC route. For this experiment, we used an anti-DENV vaccine formulation containing DENV2 New Guinea C (NGC) virus particles admixed with heat-labile toxin (LT) produced by enterotoxigenic *Escherichia coli* (ETEC) strains as an adjuvant [11,13]. As a control, mice were also immunized via the intradermal (ID) administration route with the same vaccine formulation and antigen doses. The results demonstrated that the vaccines administered via the TC route were highly immunogenic, resulting in specific anti-DENV2 antibodies (Abs), proinflammatory cytokines and protective immunity to a lethal challenge with the DENV2 NGC strain.

## 2. Materials and Methods

### 2.1. Ethics Statement

All the procedures and animal experiments carried out during this study were performed by authorized and trained people, according to the recommendations of the Committee for the Ethical Use of Laboratory Animals (CEUA) from the Institute of Biomedical Sciences of the University of São Paulo (protocol 034/2015, approved on 24 March 2015). The protocols and guidelines regarding the care and use of laboratory animals were followed in all experiments.

### 2.2. Mice

The murine model elected for this study was based on the BALB/c mouse line. Male mice from 6 to 8 weeks old were supplied by the Parasitology Department Animal House at the University of São Paulo. The animals were considered free of pathogens and routinely subjected to standard monitoring.

### 2.3. Cell Lines and Virus

*Aedes albopictus* cells of the C6/36 strain were used for viral propagation, while *Cercopithecus aethiops* kidney epithelial cells (VERO) were used for plaque assays. Briefly, VERO cells (1 × 10^5^/well) were plated in 24-well culture plates and incubated at 37 °C in a CO_2_ incubator overnight. DENV supernatant aliquots (100 μL) were 10-fold serially diluted in medium, added to the VERO cells, and incubated at 37 °C for 1 h. The viral supernatant was aspirated, and a prewarmed solution of 1% carboxymethyl cellulose medium (Synth, Diadema, Brazil) was added to each well. After 7 days incubation, the cells were fixed with 4% paraformaldehyde solution for 15 min at room temperature (RT), washed with water and stained with a 1% crystal violet solution for 10 min. The staining solution was removed and the plates washed to remove residual staining. Finally, after drying on the bench, Plaque Forming Units (PFU) were counted to obtain the virus titers, which were expressed in PFU/mL. The DENV2 New Guinea C (NGC) strain was previously isolated and adapted to infect and kill immunocompetent mice after intracranial (i.c.) administration [20].

### 2.4. Purification of the Heat Labile Toxin (LT1)

The LT was purified from a recombinant *E. coli* K12 strain transformed with a BSPKS (-) vector carrying the sequence encoding the toxin originally expressed by enterotoxigenic *E. coli* [21]. The recombinant LT1 was purified by affinity chromatography with immobilized D-galactose, as previously described [22].

### 2.5. DENV2 Harvesting and Concentration

C6/36 cells were grown in Leibovitz-15 (L-15) medium (Vitrocell, Campinas, Brazil) supplemented with 10% fetal bovine serum (FBS) (Vitrocell, Campinas, Brazil) until they reached approximately 50% confluence in plastic culture bottles (Corning, New York, NY, USA). Then, the cells were washed and infected with the DENV2 NGC at a standard MOI equal to 1, and when the cells displayed syncytial characteristics, the supernatant was harvested. The cells were centrifuged (Eppendorf, Hamburg, GER) at 3200× *g* for 15 min and then lysed by freezing at −80 °C for 5 min and melting through the liquid state three times. After being lysed, the cells were centrifuged one more time, and the supernatant containing the released virus was added to the cell culture supernatant. The whole supernatant was mixed with polyethylene glycol (PEG-6000) (Synth, Diadema, Brazil) in a proportion of 2.5 mL of PEG to 10 mL of supernatant and incubated overnight at −4 °C. After incubation, the supernatants were centrifuged at 3200× *g* for 30 min, the supernatant was discarded, and the precipitate viruses were suspended in MEM supplemented with HEPES buffer. The concentrated viruses (up to 100-fold) were titrated, and the content of total protein was also determined using an Epoch Take3 spectrophotometer (BioTek, Winooski, VT, USA) and maintained at −80 °C until being used for immunization assays.

### 2.6. DENV2 Purification

The purification of DENV2 virus particles was performed by ion exchange chromatography using the AKTA FPLC chromatograph (Amersham Pharmacia Biotech, Little Chalfont, UK) associated with a 1 mL HiTrapTM ANX (high sub) (GElifesciences, Boston, MA, USA) anion exchange column previously loaded with 60 mM sodium phosphate buffer (pH 7.2). A total of 10 mL of infected cell culture supernatant (VERO cell previously infected for 7 days with DENV2, MOI of 0.1) was used for purification. The elution procedure was carried out by applying a linear gradient from 60 to 600 mM of sodium phosphate (pH 7.2) in 15 column volumes (CV). The virus-containing fractions were combined, added with 5% sterile glycerol and stored at −80 ° C for later use.

### 2.7. Transcutaneous (TC) and Intradermal (ID) Immunization Procedures

For the ID immunizations, BALB/c mice (*n* = 5) were immunized with different vaccine formulations containing 40 µg/mL of polymyxin B, 10 or 50 µg of DENV2 NGC virus particles administered alone or with 10 µg of purified LT1. The vaccination regimen consisted of three doses given at two-week intervals (14 days). Serum samples were collected through the submandibular plexus before each immunization (days −1, 13, 27, 41). The administration was performed in the dorsal region on the back of the mice with a 25 mm gauge needle loaded with 25 μL of the vaccine formulation.

The same vaccine formulations were used in the TC immunizations. The dorsum of BALB/c mice (*n* = 5/group) was shaved with an electric clipper and depilatory cream. Before the administration of the vaccine patches, a slight abrasion with fine-grained sandpaper was applied to the shaved skin to disrupt the stratum corneum (SC). The patches were prepared with a double-sided waterproof adhesive film (Smith&Nephew, London, UK) cut in squares of approximately 2.5 cm^2^. An absorbent sterile gauze piece (approximately 1 cm^2^) was placed in the center of the adhesive film to form a pad that received the vaccine. The vaccines were placed on the back of the animals for 24 h, and bandages were applied to prevent their removal by the animals (Figure S1).

### 2.8. Analysis of Specific Antibody Responses

Mouse sera were individually tested for the presence of the virus-specific Abs by ELISAs. MaxiSorp plates (Nunc, Roskilde, DK) were coated with PBS containing 200 ng of DENV2 NGC virus particles and incubated overnight at 4 °C. The plates were blocked with 3% gelatin in PBS at 37 °C for 2 h. Serum samples were then serially diluted in 1% gelatin diluted in 0.05% PBS-Tween (PBST) and incubated at 37 °C for 1.5 h. After three washes with PBST, the plates were treated with goat anti-mouse IgG (1:6000), IgG1 (1:3000) or IgG2a (1:2000) conjugated with horseradish peroxidase (Sigma-Aldrich, San Luis, MO, USA) at 37 °C for 1.5 h. After a new wash cycle with PBST, a solution containing orthophenylenediamine dihydrochloride (OPD) (Sigma-Aldrich, San Luis, MO, USA) and H_2_O_2_ was added for a final volume of 100 µL per well. The plates containing the developer solution were maintained in the dark for 15 min, at which time, the reaction was stopped by the addition of 50 µL of 2 N H_2_SO_4_. The OD_492nm_ was measured by an Epoch Take3 spectrophotometer (BioTek, Winooski, VT, USA), and the Ab concentration values were calculated by comparison with a reference IgG preparation at defined concentrations that had been added to each plate.

Antigen avidity with serum Abs was determined with serum pools collected after the last vaccine dose was administered and carried out as previously reported [23]. After incubation with normalized concentrations of antivirus sera, plates were washed and exposed to phosphate-buffered saline (PBS)-diluted ammonium thiocyanate Sigma-Aldrich, San Luis, MO, USA ranging from 0 to 8 M. The plates were allowed to stand for 15 min at room temperature before being washed. The concentrations of the ammonium thiocyanate required to dissociate 50% of the bound Abs were determined. The percentage of binding was calculated as follows: OD_492nm_ in the presence of ammonium thiocyanate X 100/OD_492nm_ in the absence of ammonium thiocyanate. The values are expressed as percentages compared with those of the sample not subjected to the ammonium thiocyanate treatment.

### 2.9. Analysis of Cytokine Production

Mice were sacrificed, and the spleens were aseptically harvested. Splenocytes were counted after red blood cell lysis and suspended in 10% FBS/RPMI medium. Splenocytes were plated (2.5 × 10^5^ cells/well) and stimulated with 2 µg of thermally inactivated DENV2 for 96 h. The levels of secreted IL-10, TNF-α, IFN-γ, IL-4, and IL-2 were determined with a cytometric bead array (CBA) mouse Th1/Th2/Th17 cytokine kit (BD, Franklin Lakes, NJ, USA) according to the manufacturer’s protocol. The measurements were performed using BD LSRFortessa (BD, Franklin Lakes, NJ, USA), and the results were processed with system software. Three technical repeats were carried out for each animal, and the results were statistically evaluated using one-way ANOVA with Bonferroni’s post-test.

### 2.10. Lethal Challenges with the DENV2 NGC Strain

Two weeks after receiving the last vaccine dose, the immunized mice were first anesthetized with a mixture of ketamine and xylazine and subsequently challenged with 1 × 10^6^ plaque forming units (PFUs) of the NGC strain to a final volume of 50 µl administered through the intracranial (i.c.) route, as previously described [24]. These inoculated mice were monitored for 21 days for survival.

### 2.11. Statistical Analyses

All statistical analyses were calculated using Prism 5 and 6 software (GraphPad Software Inc, LA Jolla, CA, USA), and differences with *p* ≤ 0.05 were considered significant. To compare results generated in several groups, a two-way ANOVA test was applied in association with Bonferroni’s post-test. A comparison of the results generated in three groups was performed using one-way ANOVA in association with Bonferroni’s post-test. Statistical significance based on the mortality curves was determined by Mantel–Cox tests.

## 3. Results

### 3.1. Induction of DENV2-Specific Serum Antibody Responses in Mice Immunized via the TC or ID Route

**Figure 1:**
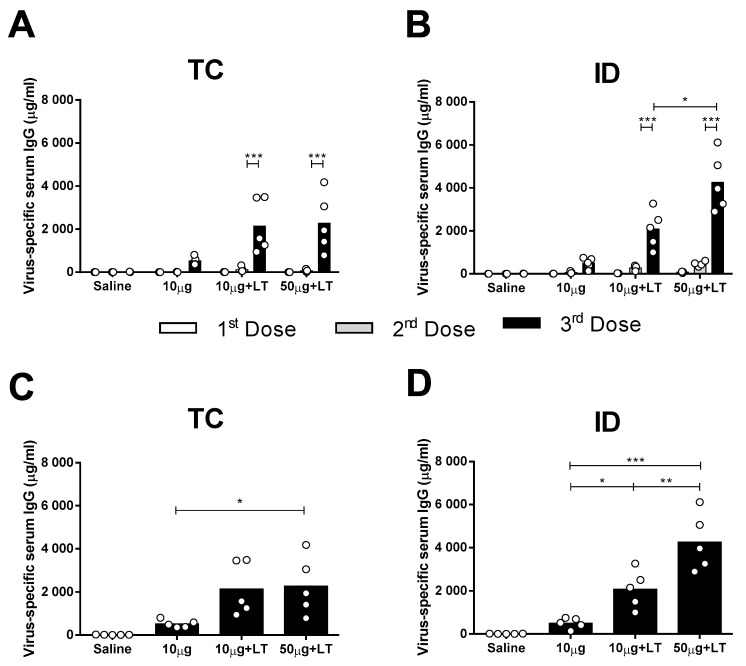
Serum IgG responses elicited in mice immunized with type 2 Dengue virus particles (DENV2) through transcutaneous (TC) or intradermal (ID) administration routes. Concentrated virus particles (10 or 50 µg protein/dose) were administered alone or with heat-labile toxin (LT) as adjuvant (10 µg/dose) in BALB/c mice (*n* = 5). The immunization protocol consisted of 3 doses administered with a two week interval. Both TC as well as ID inoculations were administered in the dorsum of the animals, as described in the Material and Methods section. (**A**,**B**) Dose-dependent anti-DENV serum IgG responses elicited in mice following administration via TC (**A**) or ID (**B**) routes. Columns represent mean IgG concentration values + SD. (**C**,**D**) Individual serum anti-DENV2 IgG responses in mice submitted to TC (**C**) or ID (**D**) immunizations measured two weeks after the last dose. Statistical analysis was performed with a two-way ANOVA test associated with Bonferroni’s post-test. * *p* < 0.05, ** *p* < 0.01, *** *p* < 0.001. Representative results of 3 independent experiments.

Male BALB/c mice received three vaccine doses via the TC or ID route with either 10 or 50 μg of concentrated DENV2 particles (NGC strain) with or without purified LT1 (Figure S1E). As shown in Figure 1, the mice inoculated with DENV2 mounted a low antigen-specific IgG response following the TC and ID immunizations (Figure 1A,B). The addition of LT1 to the vaccines significantly enhanced the DENV2-specific serum IgG responses elicited in the mice immunized via the TC or ID route, with the maximal values reached two weeks after the third vaccine dose (Figure 1A,B). Similar serum antibody responses were also measured in mice immunized virus particles purified from cell culture supernatants by anionic chromatography (Figure S2).

The results also demonstrated that the serum DENV2-specific IgG responses increased according to the amount of inoculated antigen in the ID-immunized mice but not in the mice immunized via the TC route (Figure 1A,B). The TC-immunized mice that received the higher antigen dose (50 μg) combined with LT1 showed an increased serum IgG response two weeks after the third dose (Figure 1C). The ID-immunized mice showed significant differences in DENV2-specific serum IgG responses in the presence of LT1, particularly in the group immunized with 50 µg of the tested antigen (Figure 1D). In conclusion, TC administration of the anti-DENV vaccine induced serum antigen-specific antibody responses but at lower levels than those elicited by the ID administered vaccine.

The vaccinated mice were also monitored for different physiological aspects in a search of possible vaccine-induced adverse effects (Figure S3). In general, there was no indication of significant hematological alterations or tissue damage, such as in a tissue damage marker like LDH, in mice immunized via the TC or ID route, with the exception of decreased hematocrit values and increased platelet numbers in the ID-immunized mice receiving LT1 and 50 μg of the vaccine antigen (Figure S3B). No other adverse reactions, such as weight loss or altered animal behavior, were observed in the mice in which the vaccine regimens were tested [25]. Collectively, these results indicate that the anti-DENV vaccines were safe under the experimental test conditions.

### 3.2. Characterization of the Antibody Responses in the Mice Immunized via the TC or ID Route

**Figure 2:**
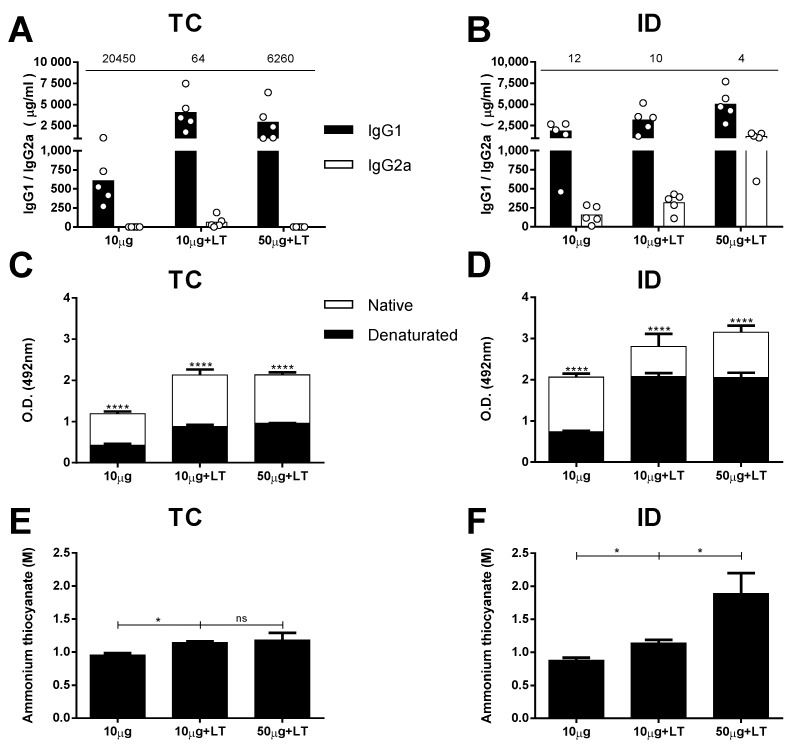
Serum antibody responses against DENV2 induced after TC or ID immunizations. DENV**-**specific serum antibody responses induced after TC and ID 3rd immunization doses, measured by ELISA with concentrated DENV particles as solid phase (*n* = 5). (**A**,**B**) Serum IgG subclass responses in mice immunized via TC (**A**) or ID (**B**) routes. Values indicated above the lines represent IgG1/IgG2a ratio, measured in each immunized group. (**C**,**D**) Binding of anti-DENV2 antibodies from serum samples of immunized mice via TC (**C**) and ID (**D**) routes to intact and heat-denatured virus particles. (**E,F**) Antigen binding affinity of anti-DENV2 antibodies induced after immunization via TC (**E**) or ID (**F**) routes. Serum samples from mice submitted to TC (**E**) or ID (**F**) immunizations were tested by ELISA using different concentrations of ammonium thiocyanate as a dissociating agent. Statistical analyses were performed by two-way ANOVA in association with Bonferroni’s post-test * *p* < 0.05, **** *p* < 0.0001. Representative results of 2 independent experiments.

We first measured the IgG-specific subclass responses in the mice subjected to different immunization regimens. The serum IgG1 subclass response prevailed in the mice immunized without adjuvant two weeks after they received the third dose. A higher IgG1/IgG2a ratio was observed among the mice immunized via the TC route compared with that of the mice immunized via the ID route (Figure 2A,B). The mice immunized with LT1 also developed a predominant IgG1 response, but those immunized with the higher antigen dose developed a more balanced IgG1/IgG2a ratio, particularly the animals immunized via the ID route (Figure 2A,B).

Aiming for an initial characterization of the antibodies capable of binding conformational epitopes in the mice immunized with the tested vaccine formulations, we measured the specificity of DENV2-specific antibodies against intact viral epitopes. For this experiment, we used heat-denatured and intact virus particles in the solid phase. As shown in Figure 2C,D, IgG was increased in the mice immunized via the TC or ID route, but significantly fewer IgG molecules were bound to the DENV particles after heat-denaturation treatment. The reduction in the anti-DENV titers for groups inoculated with LT1 adjuvant and was greater in the TC-immunized mice (55% to 58%) than it was in the ID-immunized mice (25% to 34%) (Figure S4A,B). These results suggest that a significant fraction of antibodies generated in mice immunized with intact virus particles target conformational epitopes on the virus surface. In addition, the results indicate that the proportion of antibodies targeting conformational epitopes was higher (*p* = 0.0321) in the mice immunized via the TC route than it was in the mice immunized via the parenteral route.

Finally, we measured the avidity of the DENV-specific antibodies in the mice subjected to different vaccine regimens tested. For this experiment, we followed a previously described ELISA protocol with an additional dissociation step performed with ammonium thiocyanate. Taken together, the results presented in Figure 2E,F demonstrate that the addition of LT1 steadily promoted an increase in the avidity of the antibodies to virus particles administered through both routes. However, in accordance with the measurements of the antibody titers, the amount of antigen had an impact only in the ID-immunized mice, which presented enhanced avidity after the mice received higher antigen doses (Figure 2E,F and Figure S4C,D).

### 3.3. Detection of Cytokine Secretion Profiles in the Mice Immunized with the Different Vaccines Tested

**Figure 3:**
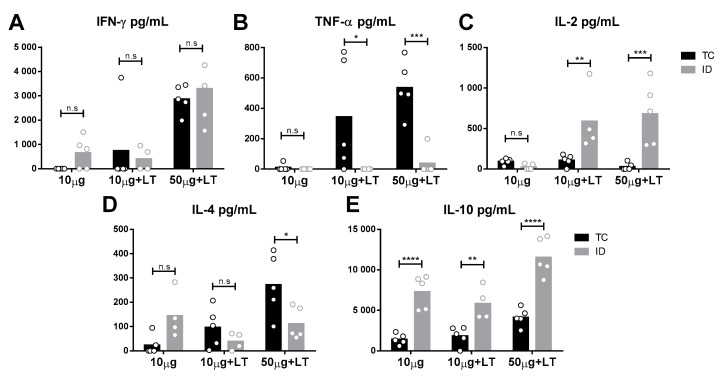
Cytokine secretion profile detected in mice submitted to TC and ID immunizations with DENV2 particles. Spleens of immunized mice (*n* = 5/ID 10 µg + LT *n* = 4) were harvested two weeks after the 3rd immunization dose and cytokines secreted by *in vitro* cultivated cells were determined according to procedures described in the Material and Methods section. (A–C) Th1 profile cytokines: interferon gamma (IFN-γ) (**A**), tumor necrosis factor alpha (TNF-α) (**B**) and interleukin 2 (IL-2) (**C**). (**D**,**E**) Th2 profile cytokines: IL-4 (**D**) and IL-10 (**E**). Statistical analysis was performed by two-way ANOVA in association with Bonferroni’s post-test * *p* < 0.05, ** *p* < 0.01, *** *p* < 0.001, **** *p* < 0.0001.

To analyze the immune responses elicited in the vaccinated mice, we assessed the cytokine secretion profile of spleen cells collected two weeks following the administration of the last vaccine dose. As indicated in Figure 3, different cytokine secretion patterns were observed according to the tested administration route. Mice immunized via the TC route showed higher tumor necrosis factor alpha (TNF-α) and interleukin 4 (IL-4) levels than shown by those immunized via the ID route (Figure 3B,D). In contrast, ID-immunized mice mounted higher IL-10 and IL-2 responses than shown by those immunized via the TC route (Figure 3C,E). Nonetheless, an increase in interferon gamma (IFN-γ) was found following immunization via both routes. Flow-cytometry assays were also performed with spleen cells after the immunization protocols prior to challenge, but no significant difference among immunized versus nonimmunized mice was observed [26].

### 3.4. Protective Immunity Conferred by the Anti-DENV2 Vaccines Inoculated via the TC or ID Route

**Figure 4:**
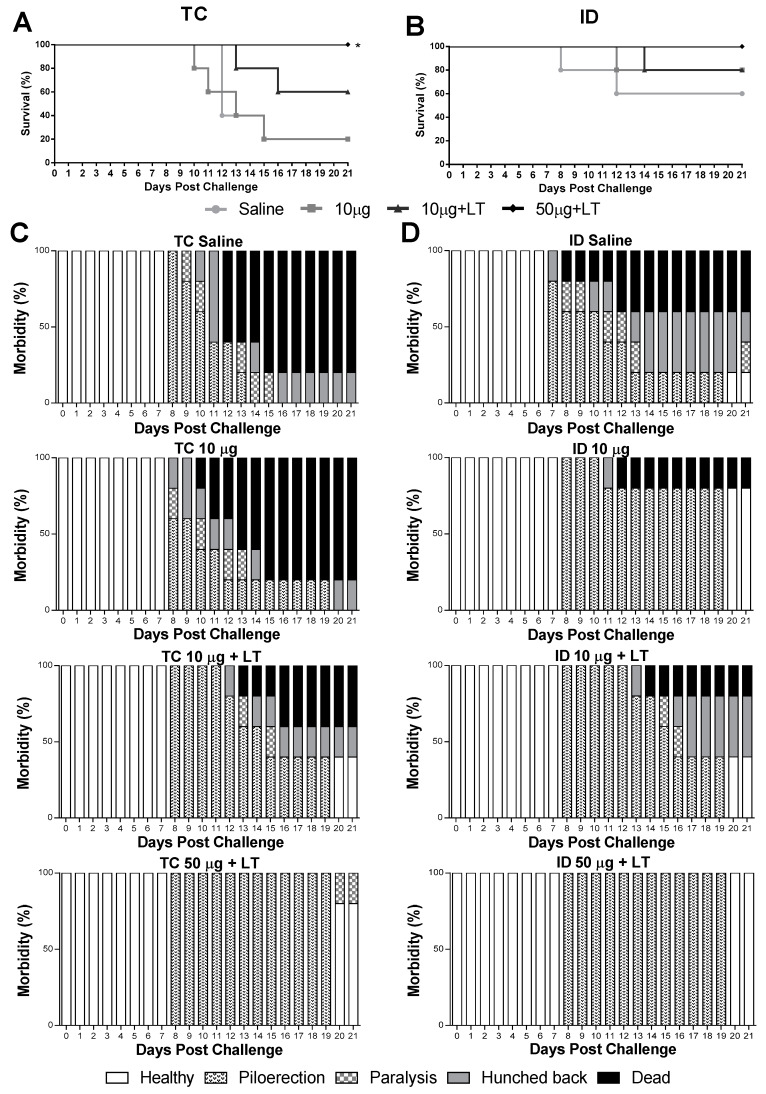
Protective immunity induced in mice after immunization via TC or ID routes with DENV2 New Guinea C (NGC). Survival and morbidity curves were mounted after inoculation of lethal intracranial (i.c.) challenge with 1 × 10^6^ NGC virus particles 2 weeks after the 3rd immunization dose (*n* = 5). (**A**,**B**) Survival curves of mice immunized via TC (**A**) or ID (**B**) routes following a lethal challenge with the DENV2 NGC strain. (**C**,**D**) Morbidity curves of mice immunized via TC (**C**) or ID (**D**) routes following the lethal challenge. Statistical analysis performed by Mantel–Cox test. * *p* < 0.05.

Mice immunized via the TC or ID route were subjected to a lethal i.c. challenge with the DENV2 NGC strain. As shown in Figure 4, the immunized mice developed partial or complete protective immunity to a lethal challenge with the DENV2 strain. In the TC-immunized mice, the conferred protection was dependent on the addition of LT1 and the antigen amount, with maximal survival values observed in the mice immunized with 50 μg of DENV2 combined with LT1 (100% survival in comparison with 20% survival in the sham-treated group) (Figure 4A). Among the ID-immunized mice, the group immunized with the higher antigen dose and LT1 were fully protected against the lethal challenge (Figure 4B). We also looked for signs of morbidity in the mice challenged with the DENV2 NGC strain (Figure 4C,D). Among the immunized mice, only those immunized with the higher antigen dose combined with LT1 displayed the maximal protection compared to that shown by the sham-treated animals (Figure 4D). At the end of the observation period, all the mice immunized via the ID route and 80% of the mice immunized via the TC route showed no signs of morbidity. Altogether, these results indicated that the administration of the tested vaccine formulation administered via either the TC or ID route induced protective immunity against DENV2.

## 4. Discussion

Despite a number of reports describing the use of TC for the delivery of different antibacterial and antivirus vaccines [5,6,7,8,14,15,16,17,18,19,27,28,29,30], thus far, only two studies addressed the performance of DENV vaccines, delivered with microneedle pads, that were tested under experimental conditions [31,32]. Here, we showed mice immunized with whole DENV2 particles through the use of adhesive patches applied on the surface of the skin and compared the results with regard to mice immunized via a parenteral route (ID). The results demonstrated that induction of dose-dependent serum virus-specific antibody responses required the incorporation of an adjuvant (LT1). In addition, although mice immunized via the TC route developed lower serum IgG responses, the protective immunity elicited in both mouse groups was similar. Such results indicate that differences in the properties of antigen-specific antibodies or activation of differential cellular immune responses contribute to the protective immunity induced in TC-immunized animals.

There are several ways to perform TC immunizations that include the use of different technologies to break the skin barrier. Microneedle devices represent an alternative to deliver the antigens right below the SC [32]. On the other hand, TC immunization may be performed with adhesive patches soaked with the antigen and applied directly on the surface skin after a gentle superficial abrasion [33]. The use of adhesive patches has several inherent advantages over parenteral routes, such as the lack of needles and pain and reduced risks of contamination, but precise determination of the amount of antigen actually delivered into the host is not possible. A precise definition of the efficiency of the TC administration route would, therefore, require comparison of immune responses and protective immunity induced after immunization with the same vaccine delivered via a parenteral route. In the present study, we performed, for the first time, such a comparison with DENV antigens and demonstrated that antigen delivered via the TC route induced similar immune responses and, more relevantly, protective immunity to DENV, thus representing an alternative for the administration of DENV vaccines. The recent report of microneedle-based TC immunization with a live-attenuated DENV vaccine further supported the use of this delivery route for the administration of live virus vaccines [32].

Previous reports demonstrated that the TC route is a safe and efficient immunization method [15,16,17,18,27,28,29,34,35]. In particular, the TC administration route prevents the toxicity of certain adjuvants, such as LT1, which may induce local and systemic side effects when delivered via parenteral routes [23,36]. Indeed, our results demonstrate that administration of DENV2 particles admixed with LT1 and delivered through the TC route led to the generation of specific antibody and cellular responses without any significant deleterious effects or local inflammatory reactions.

Studies describing the use of viral antigens administered via the TC route usually exhibit a wide range of antigen doses [7,14,19,27,37]. The antigen loads tested in the present study were designed to permit comparisons with mice immunized under the same conditions via a parenteral (ID) route. Interestingly, similar amounts of DENV Envelope (E) protein were shown to be immunogenic and protect against challenge after skin application of the microneedle device [31]. Immunization of mice with the same vaccine via two different administration routes allowed for comparisons of the impact on the antigen-specific immune responses and the induction of protective immunity. Under the test conditions, the induction of anti-DENV serum IgG responses was lower in the mice subjected to TC immunization than it was in the mice immunized via the ID route. This difference was clear after the third immunization dose and may be ascribed to the natural limitations of the naked ablated skin to efficiently deliver antigens compared with that delivered via parenteral inoculations. Despite such differences, mice immunized via the TC or ID route developed similar protective immunity after challenge with the DENV2 NGC strain. The immune responses elicited in the mice immunized via the TC route, therefore, confer a more efficient protective immunity against DENV.

Antibodies increased in the mice immunized via the TC route displayed a different reactivity toward conformation-dependent epitopes presented on the virus particles. Heat denaturation of the virus particles is expected to disrupt conformational epitopes present on structural proteins, while linear epitopes would not be affected to the same extent after similar exposure to the protein denaturation step. Indeed, results from previous studies based on human monoclonal Abs (hmAbs) generated from patients infected with DENV demonstrated that protective antibodies react preferentially to tertiary and quaternary conformational epitopes of the E protein [38]. Thus, the lower reactivity of the antibodies to the heat-denatured viruses in the TC-immunized mice may reflect differences in epitope specificity.

Antigen avidity has been reported to affect the capacity of antibodies to neutralize viruses [39]. Although mice immunized via the ID route developed antibodies with higher antigen avidity than those immunized via the TC route, particularly the mice immunized with the higher antigen concentration (50 μg/dose), the protective immunity to DENV2 was similar in both groups. Thus, antigen avidity did not seem to play a relevant role in the immune protection against DENV observed under the test conditions.

The classic protection correlate for DENV infection consists of the induction of neutralizing antibodies, but several results generated under experimental and clinical conditions indicated that the cellular arm of the immune system also plays a relevant role in the development of antiviral immunity [40,41]. Modulation of immune responses, particularly the production of Th1-related cytokines, such as IFN-γ and TNF-α, enables the activation of immune cells and control of the intracellular replication of viruses through both direct destruction of infected host cells and noncytotoxic pathways [42]. The abundant presence of intraepithelial antigen-present cells (APCs), such as Langerhans cells (LCs), capable of processing exogenous antigens and priming naïve T cells, is expected to improve the performance of vaccines delivered via this route [13]. Furthermore, a previous report showed that, in contrast to other antigen-presenting cells, few LCs are infected by DENV [43], indicating the more efficient activation of B and T lymphocytes.

In this study, the mice immunized via the TC route secreted high amounts of IFN-γ and, particularly, TNF-α. On the other hand, spleen cells collected from the ID-immunized mice secreted IFN-γ and IL-2. The production of IFN-γ and TNF as associated with the expression of CD107a was previously correlated with protection mediated by CD8^+^ T cell responses [44,45]. Moreover, IFN-γ, TNF-α and IL-2 production was correlated with subclinical DENV secondary infections [46] and may contribute to protection through multifunctional CD4^+^ T cells after vaccination [47]. The same pattern was observed in HIV patients with lower viral loads, indicating that proinflammatory responses are relevant for viral control [48].

Spleen cells collected from the mice immunized via the TC route also secreted higher amounts of IL-4 and had lower production of IL-10 in comparison with the mice immunized via the ID route. Both IL-4 and IL-10 were previously correlated with severity increases in DENV diseases [49,50,51]. IL-4 is associated with the upregulation of DENV-targeted receptors, leading to an increase in DENV infectivity in dermal APCs [52]. The production of IL-4 is modulated by DENV during infection, which explains the crescent levels after the increase in viral particle administration or adjuvant-mediated inflammation. IL-4 also induces the transformation of B cells in plasma cells [52], increasing the amount of specific antibodies produced, as observed at a level of significance in the group that received 50 µg of DENV2. IL-10 is usually associated with the suppression of pathogen-mediated inflammation and is found in high amounts in the sera of DENV-infected mice and humans [53,54]. The presence of IL-10^+^ monocytes after Yellow fever (YFV) vaccination was associated with a reduction in immune responses and immune system adverse reactions [55,56]. Accordingly, higher levels of IL-10 were expected to be observed after ID inoculation with LT1. Furthermore, IL-10^+^ CD8^+^ T cells showed higher cytolytic activity than did the IL-10^-^ CD8^+^ T cells during the control of coronavirus-induced acute encephalitis [57], suggesting possible enhancement of the survival and activation of CD8^+^ T cells during acute inflammation. Collectively, these results demonstrated that the same vaccine formulation delivered by either the ID or TC routes triggered different cellular immune responses, as indicated by the cytokine secretion pattern of total spleen cells collected from the vaccinated animals.

The experimental virus challenge model used in the present study depended on the i.c. inoculation of a mouse-adapted DENV2 strain (NGC). Although this infection route did not correspond to the infection process observed under natural conditions, the mouse-adapted virus strain represents a classic tool for the determination of protective immunity elicited in immunocompetent mice [58,59,60]. It has been demonstrated that serum antibody responses are only partial contributions to the encephalitis developed in i.c. challenge with DENV viruses [43]. Furthermore, the depletion of CD4^+^ or CD8^+^ T lymphocytes drastically reduced the vaccine-based protection observed in the encephalitis model [43,61]. Thus, the high protective status induced by the tested vaccine administered via both inoculation routes suggests the relevant role of cellular immune responses.

This study adds further experimental evidence that, similar to other virus vaccines, the TC route represents a promising route for vaccine administration capable of inducing protective immunity without the concerns associated with needle-delivered vaccines. Despite difficulties in standardizing the amount of antigen effectively delivered into the host tissues, this vaccine administration route is safer and a similarly effective alternative to parenterally administered DENV vaccines. Further studies based on preclinical models should contribute to the translation of the present results into an alternative for the clinical testing of DENV vaccines in humans.

## Supplementary Materials

The following are available online at https://www.mdpi.com/1999-4915/12/5/514/s1, Figure S1: Antigens and transcutaneous immunization procedures adopted in the study. Figure S2: Serum IgG responses in mice immunized with purified DENV2 particles. Figure S3: Hematological analyses of mice submitted to TC or ID immunizations with concentrated DENV2 particles. Figure S4: Binding of anti-DENV antibodies to viral particles following antigen heat denaturation or treatment with ammonium thiocyanate.
