# Supplementary material for: Transcutaneous Administration of Dengue Vaccines
Source: Viruses. 2020 May 6;12(5):514. doi: 10.3390/v12050514 (PMC7290698; doi:10.3390/v12050514)
Supplement: Supplementary file 1 [file viruses-12-00514-s001.pdf]

Supplementary Files

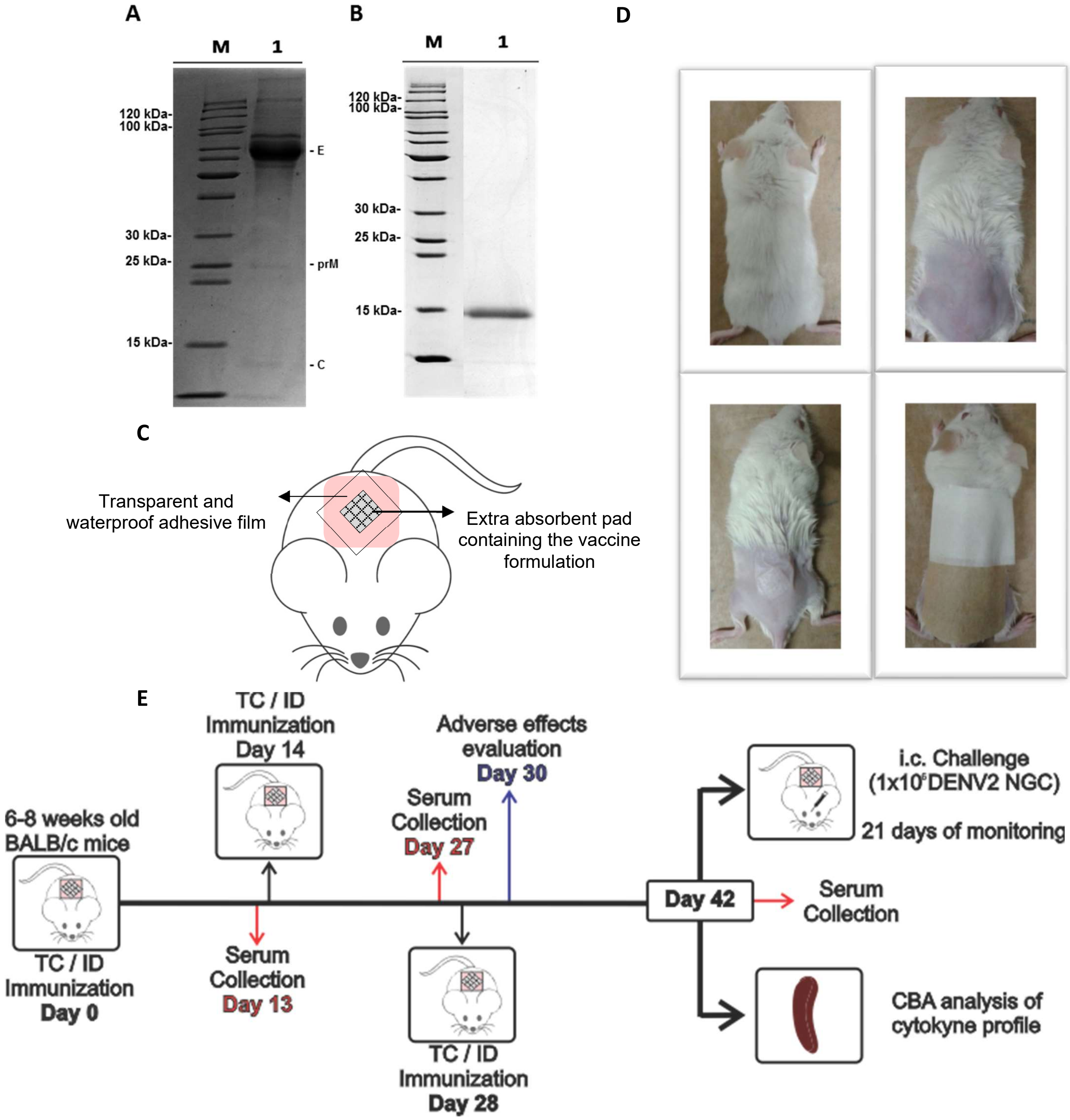

Figure S1. Antigens and transcutaneous immunization procedures adopted in the study. (A and B) Coomassie blue staining of polyacrylamide gel containing the concentrated DENV2 virus particles (A) and the purified LT used as adjuvant (B) in the immunization experiments. (C) Schematic representation of the adhesive patches containing the vaccine formulations applied via TC administration route. (D) Different steps involved in the vaccine formulation administration via TC route (from top left to low right corner). The dorsum of the animal was shaved before the vaccine pad application and subsequently wrapped with tape to avoid the vaccine pad removal by the animals. The vaccine patches were kept for 24h prior removal. (E) Flow chart comprising all manipulations and sample collections performed in the study.

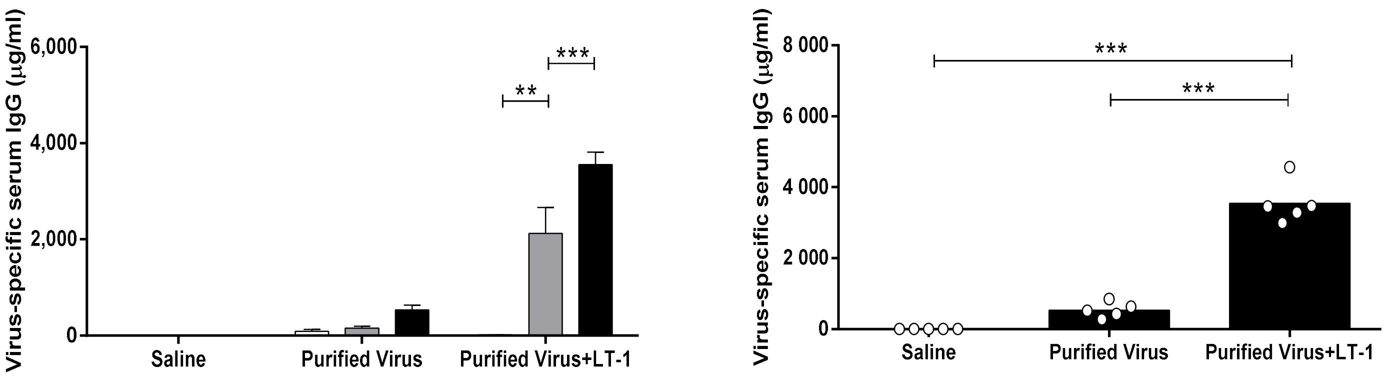

Figure S2. Serum IgG responses in mice immunized with purified DENV2 particles. BALB/c mice (n=10 per group) were immunized with viable, anionic chromatography purified, DENV2 virus particles (10µg protein/dose) co-administered with LT-1 as adjuvant (10 µg/dose). TC consisted in three doses administered on days 1, 14 and 28 for 24 h with adhesive patches. A) Dose-dependent anti-DENV IgG responses. Columns represent the mean IgG concentration values (means + SD) measured individually. B) Individual serum anti-DENV2 IgG responses in mice submitted to three TC doses (means + SD). Statistical analysis in (A) were performed by 2way ANOVA in association with Bonferroni's posttest *p<0,05. Statistical analyses in (B) were performed by One-way ANOVA in association with Bonferroni's posttest *p<0,05.

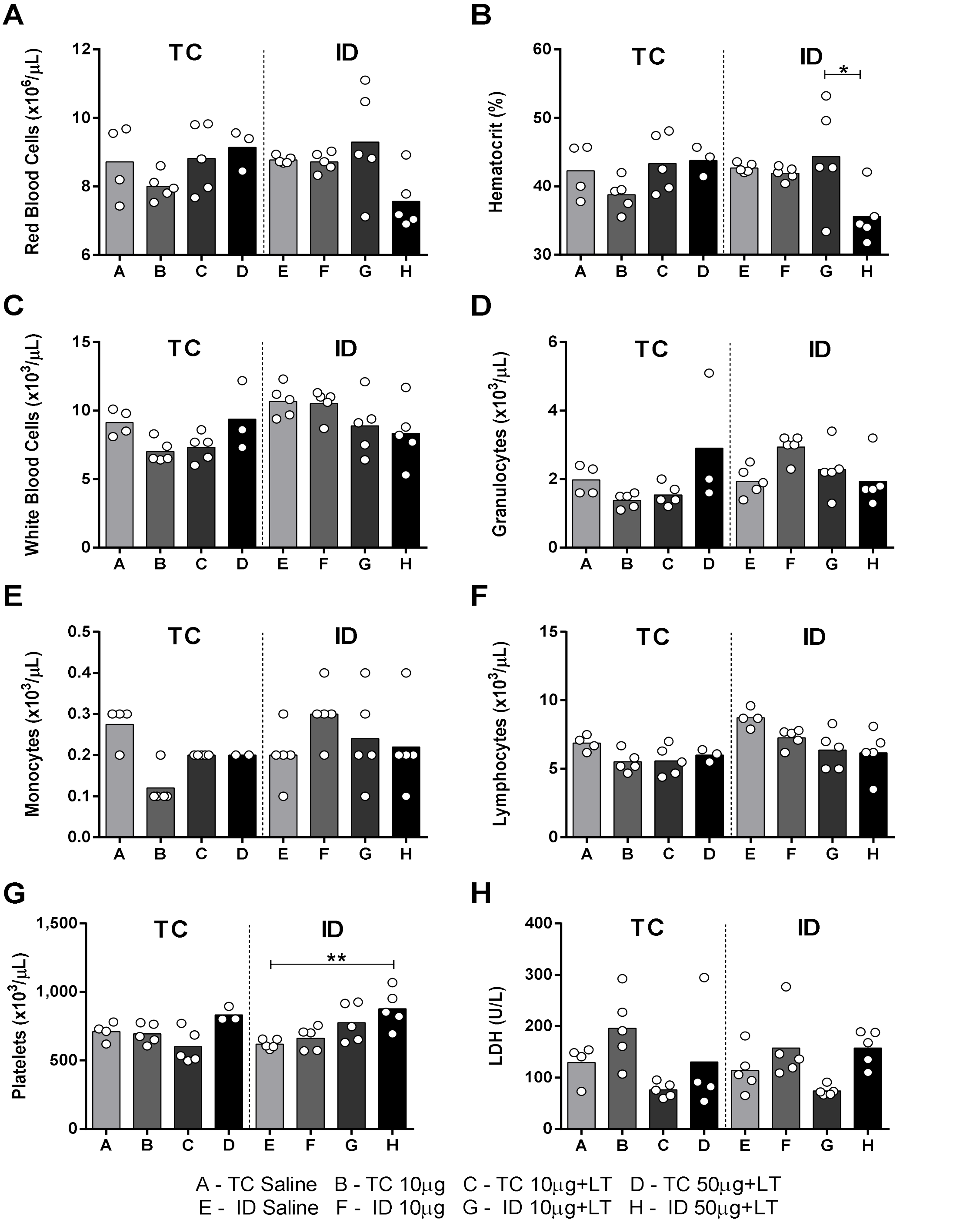

Figure S3. Hematological analyses of mice submitted to TC or ID immunizations with concentrated DENV2 particles. (A-G) Whole blood analyses of vaccinated mice in samples collected 48 h after the 3rd inoculation round. Red blood cell counting (A), hematocrit (B), white blood cells (C), granulocytes (D), monocytes (E), lymphocytes (F) and platelets (G) numbers. (H) LDH levels measured in serum samples collected 48 h after the 3rd vaccine dose. Statistical analysis was performed by 2way ANOVA in association with Bonferroni's posttest *p<0.05, **p<0.01.

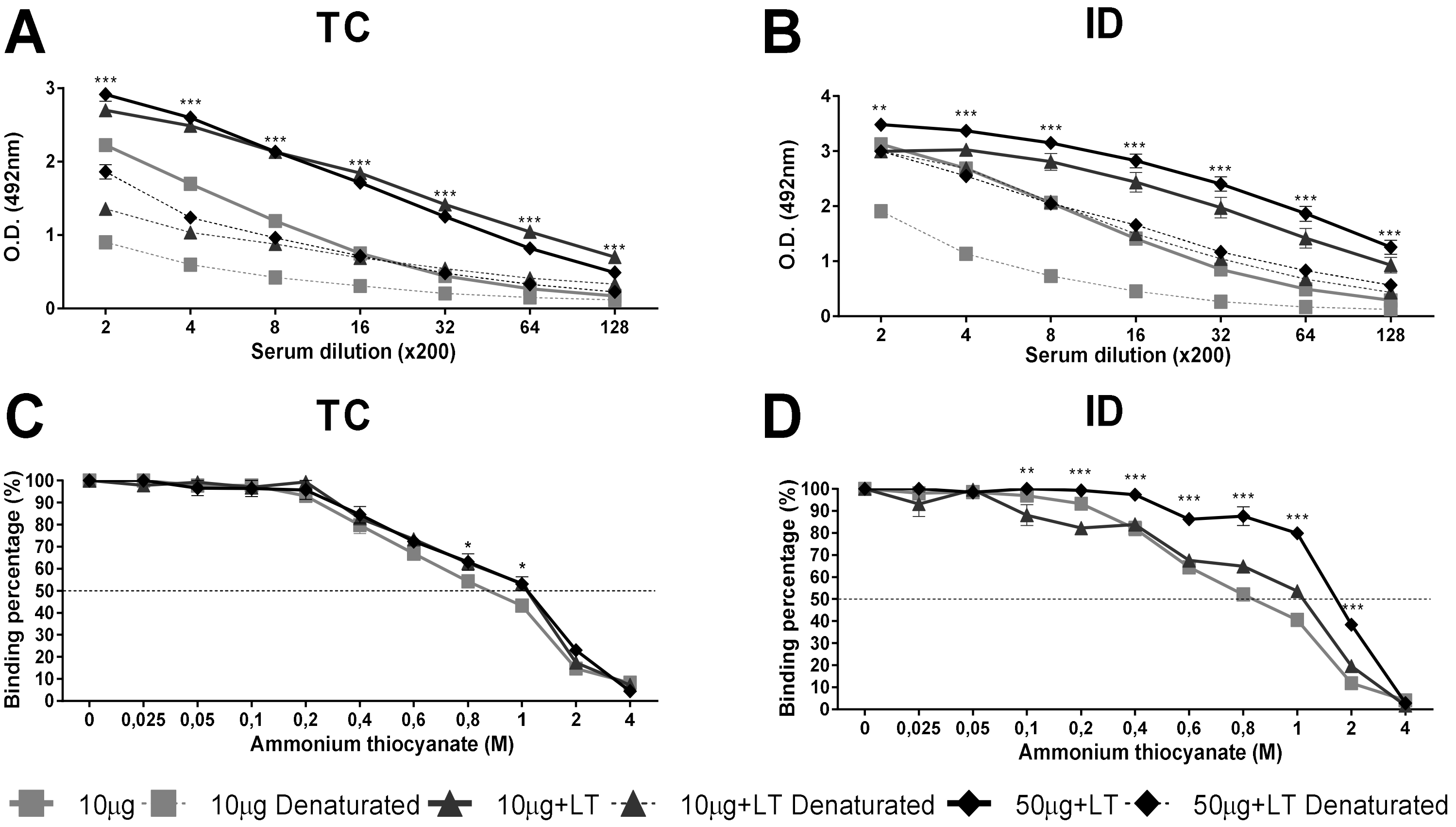

Figure S4. Binding of anti-DENV antibodies to viral particles following antigen heat denaturation or treatment with ammonium thiocyanate. (A and B) Antibodies raised in mice submitted to TC (A) or ID (B)immunizations with DENV2 particles. ELISA plates were prepared with DENV2 NGC virus particles submitted, or not, to a heat-denaturation treatment (100°C for 10 min). Solid lines represent values detected against intact DENV2 particles, while hatched lines represent values detected against DENV2 denatured particles. (C and D) Antigen affinity of anti-DENV2 antibodies raised in mice immunized via TC (C) or ID(D) routes. ELISA tests were performed with different concentrations of ammonium thiocyanate, as described in the Materials and Methods section. The dotted line indicates the point in which the amount of ammonium thiocyanate dissociates 50% of the antibodies bound to DENV2 particles. Serum samples were collected and pooled two weeks after the last vaccine dose. Statistical analysis was performed by 2way ANOVA in association with Bonferroni's posttest *$p<0.05$, **$p<0.01$***$p<0.001$.
